# Recent advances in pharmaceutical cocrystals of theophylline

**DOI:** 10.1007/s13659-024-00470-y

**Published:** 2024-09-14

**Authors:** Yanxiao Jia, Dezhi Yang, Wenwen Wang, Kun Hu, Min Yan, Li Zhang, Li Gao, Yang Lu

**Affiliations:** 1 Beijing City Key Laboratory of Polymorphic Drugs, Center of Pharmaceutical Polymorphs, Institute of Materia Medica, Chinese Academy of Medical Sciences, and Peking Union Medical College, Beijing, 100050 People’s Republic of China; 2 Prescription Laboratory of Xinjiang Traditional Uyghur Medicine, Xinjiang Institute of Traditional Uyghur Medicine, Urumqi, 830000 People’s Republic of China

**Keywords:** Pharmaceutical cocrystals, Theophylline, Physiochemical properties, Synergistic effect, Formation mechanism

## Abstract

Currently, cocrystallization is a promising strategy for tailoring the physicochemical properties of active pharmaceutical ingredients. Theophylline, an alkaloid and the most primary metabolite of caffeine, is a readily available compound found in tea and coffee. It functions primarily as a bronchodilator and respiratory stimulant, making it a mainstay treatment for lung diseases like asthma. Theophylline’s additional potential benefits, including anti-inflammatory and anticancer properties, and its possible role in neurological disorders, have garnered significant research interest. Cocrystal formation presents a viable approach to improve the physicochemical properties of theophylline and potentially mitigate its toxic effects. This review comprehensively explores several successful studies that utilized cocrystallization to favorably alter the physicochemical properties of theophylline or its CCF. Notably, cocrystals can not only enhance the solubility and bioavailability of theophylline but also exhibit synergistic effects with other APIs. The review further delves into the hydrogen bonding sites within the theophylline structure and the hydrogen bonding networks observed in cocrystal structures.

**Graphical Abstract:**

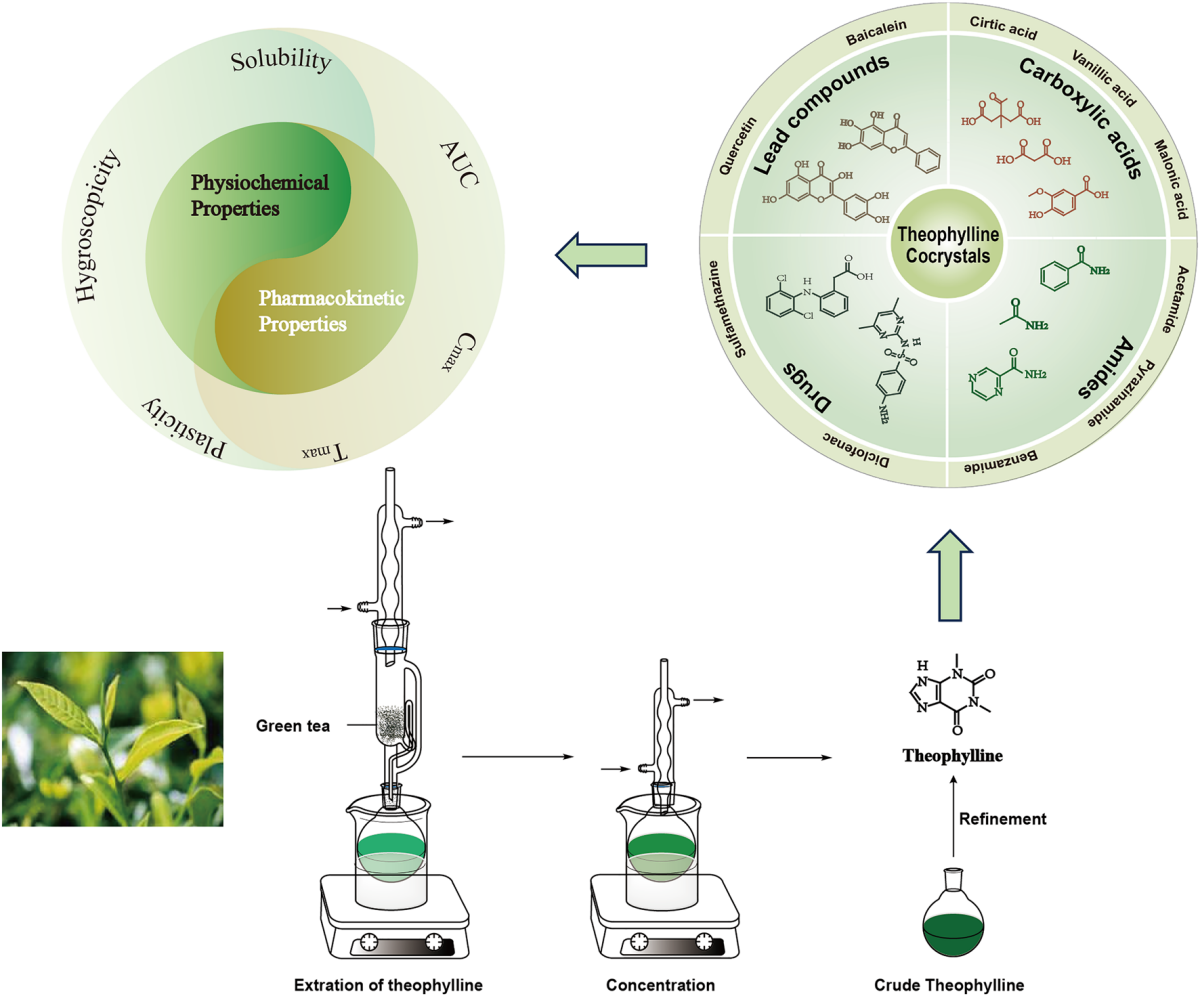

## Introduction

A cocrystal is a crystalline material formed by the ordered assembly of two or more distinct molecules in a defined stoichiometric ratio, typically stabilized by weak interactions such as hydrogen bonding and π-π stacking [1]. In the context of pharmaceuticals, cocrystals involve the combination of an active pharmaceutical ingredient (API) with a pharmaceutically acceptable cocrystal coformer (CCF) to create a new crystalline entity. Cocrystal technology offers a promising approach to enhancing the physicochemical and biopharmaceutical properties of APIs, including solubility, stability, permeability, and bioavailability, without modifying the API's core chemical structure [2].

**Fig. 1:**
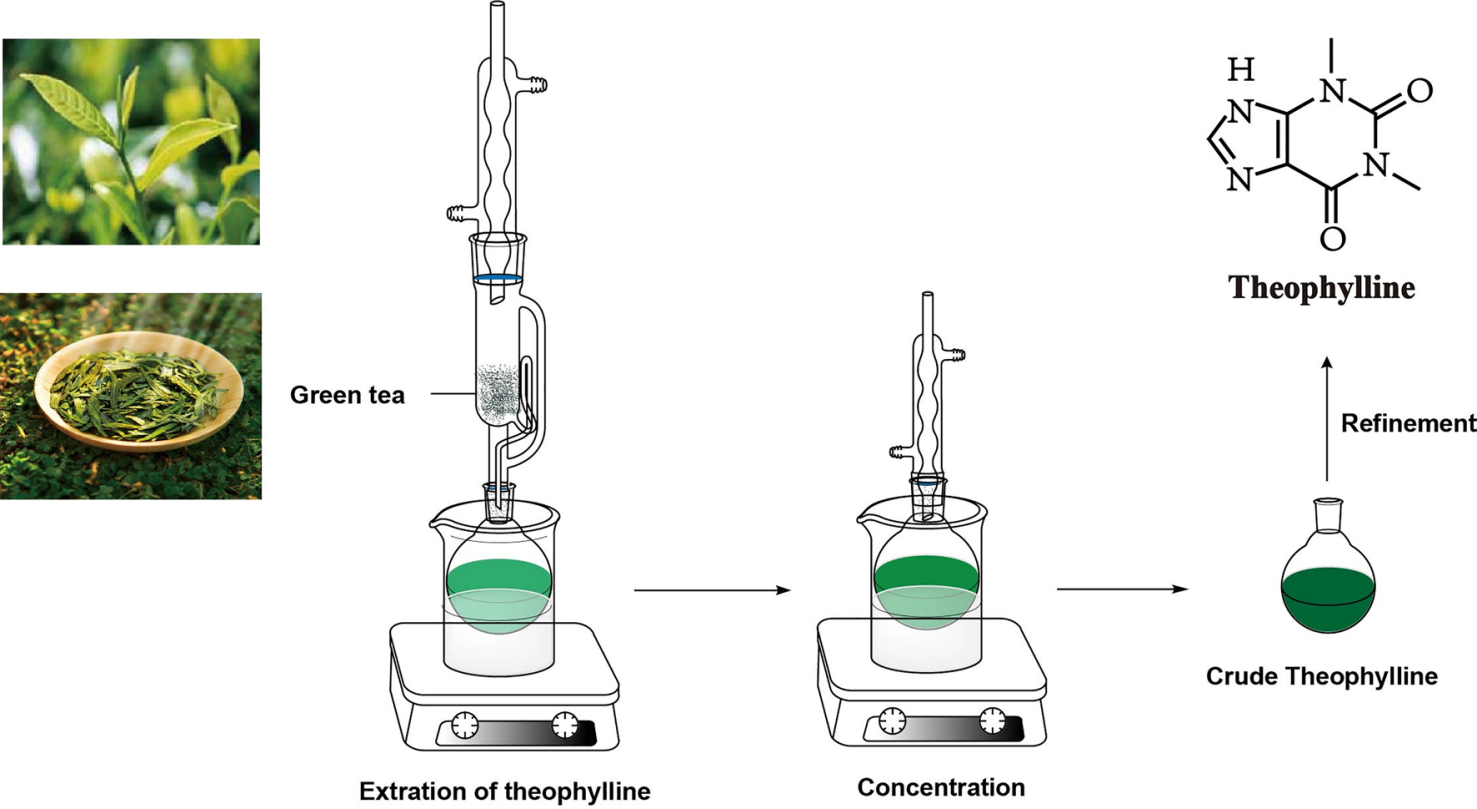
The extraction process of theophylline

Theophylline (THP), chemically designated as 1,3-dimethyl-3,7-dihydro-1H-purine-2,6-dione, is a biologically significant N-methylated xanthine found in tea, coffee, and cocoa beans [3]. THP can be obtained through direct extraction and concentration from natural sources like green tea or synthesized chemically [4, 5]. Figure 1 exhibits the molecular structure of THP and its extraction process. THP exhibits weak acidic and basic properties with equivalent pK_a_ and pK_b_ values of 8.6 and 11.5, respectively [6]. Theophylline has served as a mainstay treatment for respiratory conditions like asthma and chronic obstructive pulmonary disease (COPD) for nearly nine decades, primarily due to its bronchodilator activity. Additionally, its well-established antitussive action makes it a valuable option for managing cough in COPD patients. THP's bronchodilator and antitussive effects stem from its weak and non-selective phosphodiesterase (PDE) inhibition, particularly targeting PDE-3 and PDE-4 [7]. Five anhydrous polymorphs of THP have been identified to date, with applications in treating cardiac illness and asthma. Seton et al. [8] characterized Forms I to IV in a recent publication. Roy et al. [9] subsequently identified and synthesized Form V through crystallization from supercritical CO_2_ and methanol, respectively.

The unique crystal structure of a cocrystal often leads to physicochemical properties that differ significantly from those of its starting materials. This characteristic makes pharmaceutical cocrystals valuable tools for the pharmaceutical industry. By employing cocrystallization, researchers can tailor the physicochemical properties of APIs, including solubility, dissolution rate, bioavailability, hygroscopicity, compressibility, tabletability, and stability, without modifying the core chemical structure of the API itself [10, 11]. This offers a distinct advantage compared to other strategies like salt formation, solvates, and polymorphs. Pharmaceutical cocrystals can be prepared using various techniques, broadly classified into solution-based and solid-based methods. Solution-based methods, such as solvent evaporation, anti-solvent precipitation, cooling crystallization, reaction cocrystallization, and slurry conversion, involve manipulating solutions containing the API and CCF. Solid-based methods, on the other hand, involve processing the API and CCF in the solid state. Examples of solid-based methods include contact cocrystallization, neat grinding, liquid-assisted grinding, and melt crystallization [12]. Notably, all these methods can be potentially employed to prepare THP cocrystals.

In this review, we explored the prevalent types of theophylline cocrystals. We analyzed the intermolecular interactions that govern the structures of these cocrystals. Subsequently, the findings of studies investigating the formation of cocrystals were presented to improve the physicochemical and biopharmaceutical properties of THP or its CCF. These properties include aspects like plasticity, solubility, hygroscopicity, and bioavailability.

## Cocrystals of THP

**Fig. 2:**
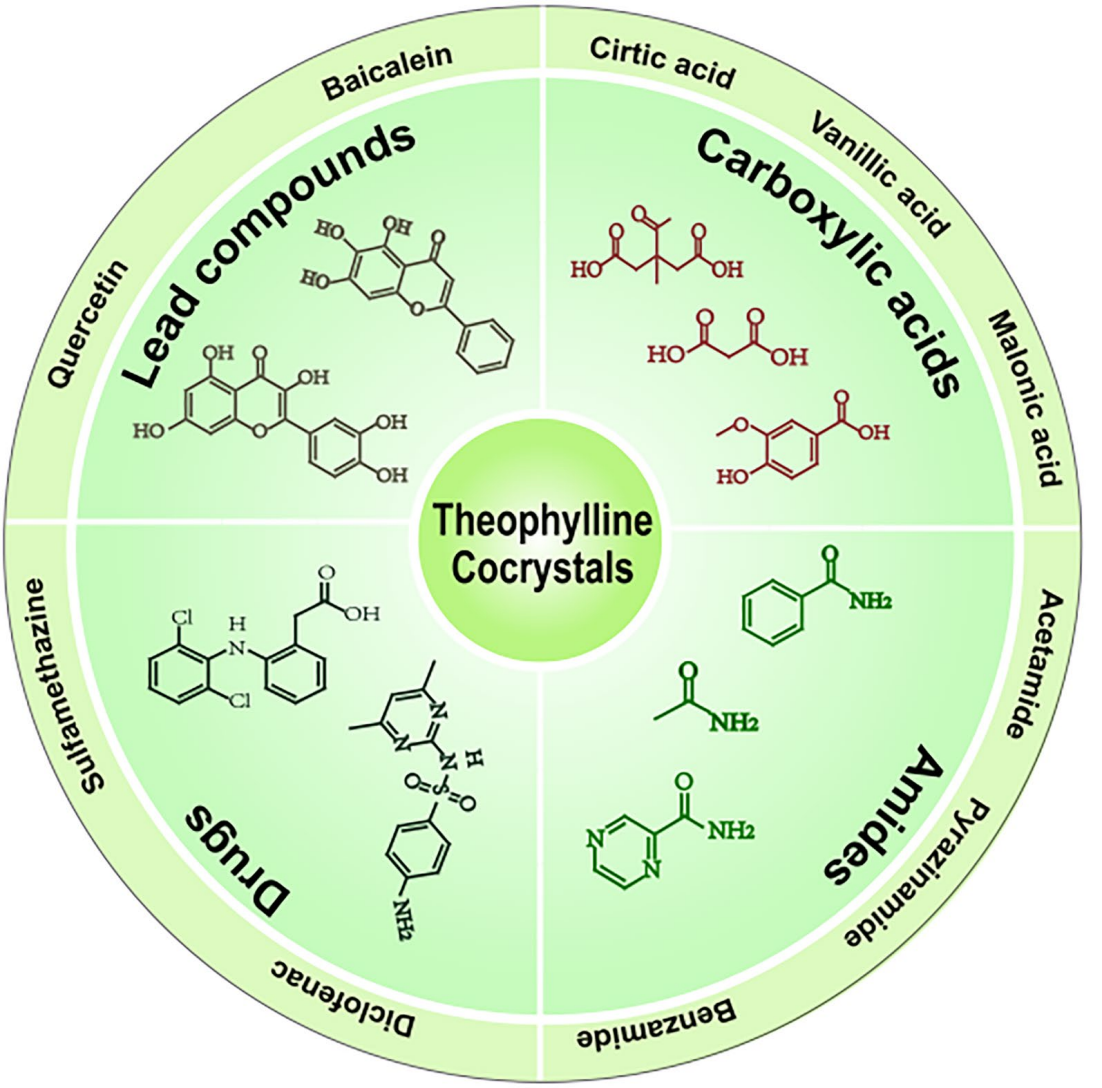
The distinct classes of theophylline cocrystals

THP, a well-studied compound, has served as a versatile building block in the creation of diverse cocrystals. It can function as either the API or the CCF within these structures. As illustrated in Fig. 1, THP possesses an imidazole proton, two carbonyl groups, and an imidazole nitrogen, offering one hydrogen-donor and three hydrogen-acceptor sites. These functional groups play a crucial role in facilitating hydrogen bonding, which is essential for the synthesis of a variety of cocrystals with different CCF [13]. Numerous studies have documented the successful application of cocrystallization to enhance specific physicochemical properties of THP, such as solubility and hygroscopicity [7, 13–23]. The substances combined with THP to form cocrystals exhibit a remarkable range of properties. CCF can be acidic, alkaline, or neutral molecules. They can include other APIs with distinct pharmaceutical activities and clinical uses, or common chemical substances without independent medicinal applications. Therefore, the structural characteristics of THP and the diverse nature of CCFs contribute to the existence of various THP cocrystal types. Consequently, the crystal structures of these cocrystals differ in terms of their hydrogen bonding patterns. Based on the functional and structural properties of CCFs, THP cocrystals can be broadly categorized as THP-carboxylic acid cocrystals [18, 24–31], THP-amide cocrystals [32], THP-lead compounds cocrystals [7, 33–38], THP-drug cocrystals [13, 39–54], THP-metal coordination cocrystals [55–66], and others. Figure 2 exhibits some different THP’s cocrystals classified based on the nature of their CCFs.

While THP is classified as a Biopharmaceutics Classification System (BCS) Class I drug, demonstrating favorable solubility and permeability, its therapeutic application in asthma and COPD is limited by potential side effects. Sustained-release formulations offer a strategy to potentially mitigate these side effects by controlling the release rate of THP. Notably, the combination of THP with metal coordination complexes has emerged as a promising approach for achieving this goal. Beyond the aforementioned classification scheme, THP can also form coordination complexes with various metal ions, leading to improved properties. Several metal coordination compounds have been successfully cocrystallized with THP, including those containing copper (Cu), silver (Ag), zinc (Zn), sodium (Na), cobalt (Co), magnesium (Mg), calcium (Ca), rhodium (Rh), and platinum (Pt). Studies by Zi-Li Xu et al. [61], demonstrated the synthesis of two novel cocrystals: [Mg(THP)_2_(H_2_O)_4_] and [Ca(THP)_2_(H_2_O)_4_]·(TPL)_2_. In vitro release experiments were conducted to compare the release profiles of THP from tablets containing the two cocrystals and pure THP. The experiments were performed at 37 °C in deionized water and phosphate-buffered saline (PBS, pH7.4). The results revealed a significantly slower release rate of THP from the cocrystal tablets compared to pure THP. For instance, in the deionized water medium, the release of pure THP reached 95% within 70 min, whereas the release of THP from [Mg(THP)_2_(H_2_O)_4_] and [Ca(THP)_2_ (H_2_O)_4_]·(THP)_2_ reached only 71% and 34%, respectively. These findings suggest that the presence of coordination bonds and hydrogen bonding interactions within the metal-THP complexes effectively controls drug release, with the release rate being influenced by the specific metal involved. Importantly, cytotoxicity assays using the MTT method revealed no significant cytotoxicity of the two cocrystals towards NIH-3T3 cells, in contrast to the established cytotoxicity of THP itself.

The release profile of THP can be further modulated by the nature of the second ligand present in THP-metal cocrystals. For instance, a study by Benyong Lou and Fengdan He [58], investigated three cocrystals formed between zinc (II) and THP, each complexed with a different biocompatible organic acid: acetic acid, benzoic acid, and nicotinic acid. The resulting cocrystals, [Zn_2_(THP)_2_(ac)(OH)]_n_, [Zn_2_(THP)_2_ (bz)(OH)]_n_, and [Zn(THP)(nit)_∞_, respectively, exhibited distinct THP release patterns in pure water at 37°C. The cumulative release of THP after 10 h varied amongst the cocrystals (36.5%, 55.5%, and 36.5%, respectively), and they also achieved their maximum release at different time points (68% after 60 h, 70% after 48 h, and 50% after 60 h, respectively). These findings highlight that the selection of various biocompatible organic compounds as secondary ligands can influence the design of metal-THP cocrystals with tailored THP release characteristics in aqueous environments.

## Structures of THP cocrystals

The formation of cocrystals is primarily driven by the establishment of supramolecular interactions, with hydrogen bonding networks being the most prominent [67]. While π-π interactions can also contribute to structural organization, they generally exhibit a weaker influence compared to hydrogen bonds [68]. Due to their directionality and inherent strength, hydrogen bonds are frequently exploited in cocrystal design. In the case of THP cocrystals, hydrogen bonding plays the primary role in their formation. Therefore, understanding general hydrogen bonding principles is crucial for predicting cocrystal formation driven by this interaction. Systematic studies of cocrystals have revealed two key observations: (1) all suitable hydrogen bond donors and acceptors tend to be involved in bonding, and (2) the strongest hydrogen bond donor in a crystal structure typically interacts with the strongest acceptor. This "best-donor-best-acceptor" principle holds significant weight in the design of hydrogen bonding interactions [18]. Notably, the presence of strong C=O and N–H sites for hydrogen bonding in THP contributes to its exceptional cocrystal formation capability.

The formation of hydrogen bonds with CCFs offers a strategy to tailor the physicochemical properties of THP [37]. Studies have shown that cocrystallization with various acidic CCF can effectively inhibit THP's hygroscopic nature. This inhibition is attributed to the formation of specific hydrogen bonds: O–H (carboxyl)···N (imidazole) and N–H (imidazole)···O=C (carboxyl) [13, 18]. The improved stability of THP-acid cocrystal formulations against higher relative humidity can be further explained by the replacement of weaker hydrogen bonds. In the presence of imidazole nitrogen acting as a hydrogen bond acceptor, THP readily interacts with atmospheric moisture to form hydrates. This phenomenon leads to the substitution of the weaker hydroxyl O–H···N imidazole synthon with the stronger COOH···N imidazole synthon [69].

THP possesses one hydrogen bond donor (N–H (imidazole)) and three hydrogen bond acceptors (O=C (carboxyl), imidazole nitrogen). During cocrystal formation, all or some of these sites can participate in hydrogen bond formation. In THP-acid cocrystals, the N–H (imidazole) and O=C (carboxyl) groups of THP consistently form hydrogen bonds with the O=C and O–H moieties of the CCF's carboxylic acid group, as exemplified in THP-benzoic acid [29], THP-Aspirin [13], and THP- (4-Aminobenzoic acid) [14] cocrystals. Conversely, in (THP)_2_- (d-tartaric acid) and (THP)_2_- (l-tartaric acid) cocrystals [70], only the THP's imidazole nitrogen forms hydrogen bonds with the carboxylic acid's hydroxyl group. The THP- (citric acid) cocrystal [24], presents a unique case where solely the O=C group of THP acts as a hydrogen bond acceptor, interacting with the hydroxyl groups in citric acid. Notably, hydrogen bonding can also occur between THP molecules or CCF molecules, depending on the stoichiometry within the asymmetric unit. In the THP-quercetin cocrystal hydrate, two THP molecules connect through N–H (imidazole)···O=C hydrogen bonds. One THP molecule interacts with quercetin via an O–H···N (imidazole) hydrogen bond, while the two quercetin molecules form an O–H (carboxyl)···O=C hydrogen bond. Additionally, water molecules contribute to the connection between two quercetin molecules through O–H···O hydrogen bonds [7].

**Fig. 3:**
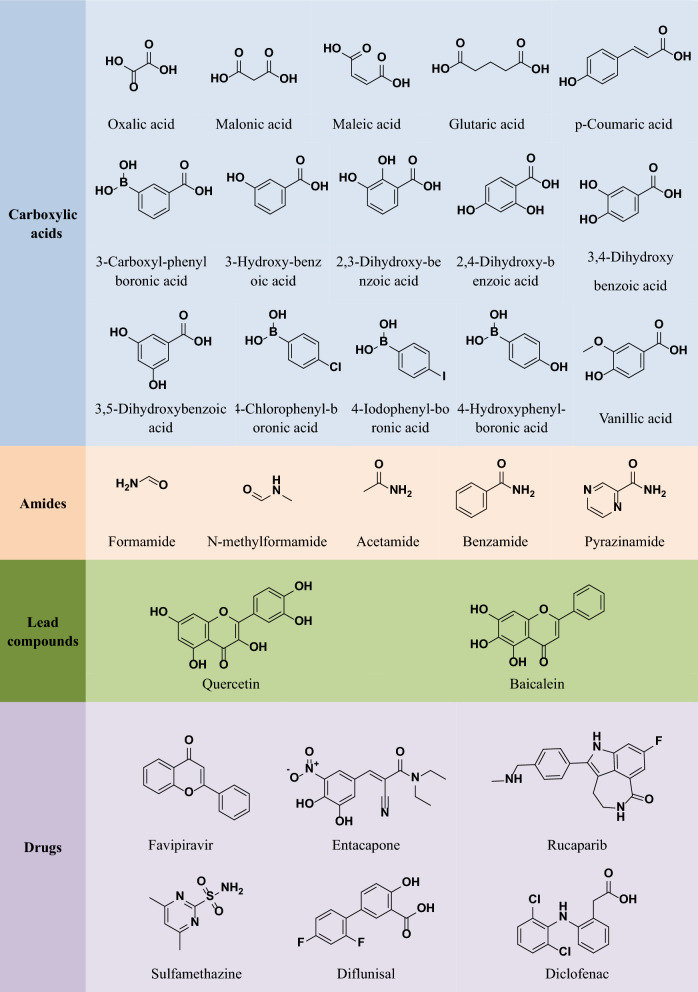
The structures of CCFs used for cocrystallization with THP

Numerous cocrystals of THP with various CCFs have been reported over the years. Table 1 exhibits the relevant crystallographic data of some THP cocrystals and Fig. 3 exhibits the structures of all the compounds listed in Table 1 that are capable of forming cocrystals with THP [7, 18, 43, 46, 64, 71].

**Table 1:** The crystallographic data of some THP cocrystals

Cocrystal	Chemical formula	Formula weight	Space group	a (Å) b (Å) c (Å)	α (°) β (°) γ (°)	*Z*
2THP-Oxalic acid (2:1)	(C_7_H_8_N_4_O_2_)_2_· C_2_H_2_O_4_	450.38	*P*2_1_/*c*	5.821 (1) 16.609 (3) 9.806 (2)	90 99.83 (3) 90	2
THP-Malonic acid (1:1)	C_7_H_8_N_4_O_2_· C_3_H_4_O_4_	284.24	*C*2/*c*	17.188 (4) 8.388 (2) 17.629 (4)	90 105.684 (2) 90	8
THP-Maleic acid (1:1)	C_7_H_8_N_4_O_2_· C_4_H_4_O_4_	296.25	$P\overline{1 }$	7.971 (16) 8.613 (17) 10.665 (2)	69.55 (3) 72.53 (3) 71.24 (3)	2
THP-Glutaric acid (1:1)	C_7_H_8_N_4_O_2_· C_5_H_8_O_4_	312.29	*P*2_1_/*c*	9.599 (2) 19.897 (4) 15.326 (4)	90 107.885 (1) 90	4
THP-Favipiravir (1:1)	C_7_H_8_N_4_O_2_· C_5_H_4_FN_3_O_2_	337.29	*P*2_1_/*n*	13.407 (2) 7.469 (12) 14.869 (2)	90 107.834 (5) 90	4
THP-Entacapone- H_2_O (1:1:1)	C_7_H_8_N_4_O_2_· C_12_H_10_N_3_O_5_· H_2_O	474.48	$P\overline{1 }$	7.102 (2) 7.335 (2) 22.712 (7)	95.075 (2) 90.438 (2) 101.915 (2)	2
THP-Rucaparib H_2_O (1:1:1)	C_7_H_8_N_4_O_2_· C_19_H_18_FN_3_O· (H_2_O)	521.52	$P\overline{1 }$	9.300 (3) 10.889 (4) 12.182 (4)	74.807 (1) 73.459 (1) 72.820 (1)	2
THP-Quercetin- 2H_2_O (1:1:2)	C_7_H_8_N_4_O_2_· C_15_H_10_O_7_· (H_2_O)_2_	982.82	*P*2_1_/*c*	7.300 (4) 32.164 (17) 17.733 (10)	90 96.995 (2) 90	4
THP-p-Coumaric I acid (1:1)	C_7_H_8_N_4_O_2_· C_9_H_8_O_3_	344.33	$P\overline{1 }$	7.707 (6) 14.271 (10) 14.704 (10)	74.327 (4) 82.294 (5) 83.154 (5)	4
THP-p-Coumaric II acid (1:1)	C_7_H_8_N_4_O_2_· C_9_H_8_O_3_	344.33	*P*2_1_/*n*	6.920 (11) 26.801 (4) 8.698 (12)	90 108.027 (6) 90	4
THP-3-Carboxylphenylboronic acid (1:1)	C_7_H_8_N_4_O_2_· C_7_H_7_BO_4_	346.11	*P*2_1_/*c*	13.185 (4) 9.189 (3) 3.287 (4)	90 109.04 (3) 90	4
THP-3-Hydroxybenzoic acid (1:1)	C_7_H_8_N_4_O_2_· C_7_H_6_O_3_	318.29	$P\overline{1 }$	7.683 (2) 8.191 (2) 23.307 (6)	98.202 (5) 92.589 (5) 103.123 (4)	4
THP-2,3-Dihydroxybenzoic acid (1:1)	C_7_H_8_N_4_O_2_· C_7_H_6_O_4_	334.29	*P*2 _1_/*c*	15.327 (7) 6.792 (3) 14.041 (6)	90 106.297 (4) 90	4
THP-2,4-Dihydroxybenzoic acid (1:1)	C_7_H_8_N_4_O_2_· C_7_H_6_O_4_	334.29	*P*2_1_/*c*	12.565 (4) 7.894 (3) 14.528 (5)	90 98.840 (6) 90	4
THP-3,4-Dihydroxybenzoic acid (1:1)	C_7_H_8_N_4_O_2_· C_7_H_6_O_4_	334.29	$P\overline{1 }$	8.069 (12) 8.578 (13) 11.558 (17)	103.147 (2) 104.970 (2) 105.358 (2)	2
THP-3,5-Dihydroxybenzoic acid (1:1)	C_7_H_8_N_4_O_2_· C_7_H_6_O_4_	334.29	$P\overline{1 }$	7.225 (17) 8.000 (19) 12.650 (3)	81.240 (4) 85.071 (4) 82.509 (4)	2
THP-4-Chlorophenylboronic acid (1:1)	C_7_H_8_N_4_O_2_· C_6_H_6_BClO_2_	336.54	$P\overline{1 }$	7.510 (1) 9.471 (2) 11.878 (2)	85.63 (3) 79.47 (4) 68.96 (3)	2
THP-4-Iodophenylboronic acid (1:1)	C_7_H_8_N_4_O_2_· C_6_H_6_BIO_2_	427.99	$P\overline{1 }$	7.475 (1) 9.623 (2) 11.957 (3)	87.25 (1) 80.94 (1) 71.63 (1)	2
THP-4-Hydroxyphenylboronic acid (1:1)	C_7_H_8_N_4_O_2_· C_6_H_7_BO_3_	318.10	*P*2_1_/*c*	12.858 (3) 8.215 (2) 14.199 (3)	90 98.03 (4) 90	4
2THP-Baicalein-H_2_O (2:1:1)	(C_7_H_8_N_4_O_2_)_2_· C_15_H_10_O_5_· (H_2_O)_3_	684.56	*P*2_1_	6.729 (5) 14.923 (9) 15.105 (10)	90 97.145 (4) 90	2
THP- Formamide I (1:1)	C_7_H_8_N_4_O_2_· CH_3_NO	225.20	*P*2_1_/m	8.731 (3) 6.658 (3) 8.900 (4)	90 98.546 (2) 90	2
THP-Formamide II (1:1)	C_7_H_8_N_4_O_2_· CH_3_NO	225.20	$P\overline{1 }$	6.606 (13) 8.716 (17) 8.884 (18)	81.34 (3) 87.63 (3) 87.47 (3)	2
THP-N-methylformamide (1:1)	C_7_H_8_N_4_O_2_· C_2_H_5_NO	239.23	$P\overline{1 }$	6.632 (3) 8.791 (4) 9.596 (4)	92.441 (2) 92.929 (2) 90.609 (2)	2
THP-Acetamide (1:1)	C_7_H_8_N_4_O_2_· C_2_H_5_NO	239.23	$P\overline{1 }$	7.655 (13) 8.349 (14) 8.954 (16)	90.552 (8) 91.339 (11) 110.177 (12)	2
THP-Benzamide (1:1)	C_7_H_8_N_4_O_2_· C_7_H_7_NO	301.30	*P*2_1_/*c*	7.528 (2) 13.389 (4) 13.856 (4)	90 91.486 (2) 90	4
THP-Pyrazinamide I (1:1)	C_7_H_8_N_4_O_2_· C_5_H_5_N_3_O	303.27	*Pna2*_*1*_	13.455 (2) 13.288 (2) 7.6215 (4)	90 90 90	4
THP-Pyrazinamide II (1:1)	C_7_H_8_N_4_O_2_· C_5_H_5_N_3_O	303.27	$P\overline{1 }$	7.480 (2) 7.696 (2) 12.703 (4)	86.113 (2) 75.930 (2) 68.995 (2)	2
THP-2Sulfamethazine (1:2)	C_7_H_8_N_4_O_2_· (C_12_H_14_N_4_O_2_S)_2_	736.84	*P*2_1_/*c*	15.883 (6) 8.100 (3) 27.791 (10)	90 91.835 (2) 90	4
THP-2Vanillic acid (1:2)	C_7_H_8_N_4_O_2_· (C_8_H_8_O_4_)_2_	515.45	*P*2_1_/*c*	11.274 (6) 15.771 (10) 13.375 (8)	90 90.572 (2) 90	4
THP-Diflunisal (1:1)	C_7_H_8_N_4_O_2_· C_14_H_11_Cl_2_NO_2_	430.36	$P\overline{1 }$	7.525 (3) 12.138 (4) 12.231 (4)	73.505 (6) 80.547 (6) 80.693 (6)	2
THP-Diclofenac (1:1)	C_7_H_8_N_4_O_2_· C_13_H_8_F_2_O_3_	476.31	*P*2_1_/*c*	7.073 (4) 35.014 (2) 7.686 (4)	90 100.827 (1) 90	4

## Functions of THP cocrystals

As mentioned above, THP serves as a well-established example of an API with applications in treating respiratory conditions like asthma. However, its clinical use necessitates careful consideration due to limitations such as a narrow therapeutic window [72, 73]. In addition, THP undergoes metabolism by the human cytochrome P450 system in the liver. Consequently, frequent administration of high THP dosages in clinical settings can lead to hepatic toxicities [14].

Cocrystallization is a widely employed technique with the potential to modify the physicochemical and biopharmaceutical properties of various substances. Numerous studies have investigated cocrystals to evaluate the extent to which solubility and other properties are altered upon cocrystal formation. For instance, the cocrystal of saccharin and carbamazepine exhibits improved solubility compared to the poorly soluble carbamazepine alone [74]. Moreover, cocrystallization can be a valuable strategy for enhancing the physicochemical characteristics of ionizable APIs. Haeria et al. [75], demonstrated that the aqueous solubility of aspirin significantly increased upon cocrystallization with a nicotinamide CCF. This improvement in solubility translated to a higher likelihood of patient absorption of the permeable aspirin in solution. In a similar vein, cocrystallization offers a promising approach for improving the physicochemical and biopharmaceutical properties of theophylline or its cocrystal CCF, as evidenced by numerous reported studies.

### Plasticity

The plastic properties of organic crystals significantly impact the processability of pharmaceutical substances during various stages, including granulation, compression, and quality control. The degree of plasticity exhibited by pharmaceutical substances can vary across their polymorphs due to several factors, such as the arrangement of atoms, the presence of solvate molecules, and the nature of intermolecular interactions. Stephen Chan et al. [76] experimentally investigated the distinct plastic behaviors of form II anhydrous theophylline (THPa) and its monohydrate (THPm), elucidating the molecular mechanisms responsible for plasticity in these different crystal forms. Their study revealed that plastic deformation occurred in THPa crystals upon application of a load using a metallic needle and forceps, while THPm crystals exhibited elastic deformation, regaining their original shape upon removal of the applied force. These findings suggest that the plasticity observed in THPa, and potentially other simple aromatic compounds, is linked to the energy barrier associated with strain-induced chemical rearrangement. Conversely, the presence of water molecules in THPm disrupts the crystal packing arrangement, hindering the underlying mechanism that facilitates flexibility. Therefore, the introduction of hydrates may not always be a viable strategy for engineering the desired mechanical behavior of organic crystalline materials.

### Solubility

Solubility is a critical parameter in pharmaceutical research and development [77], as low solubility often translates to poor in vivo bioavailability. Cocrystallization has emerged as a promising strategy to effectively improve the water solubility of THP. This improvement in THP solubility can lead to increased bioavailability, potentially allowing for a reduction in the administered dose. Consequently, this approach may contribute to alleviating the potential toxic and adverse effects associated with THP. However, it is important to note that cocrystallization can also influence the solubility of the CCF. In certain cases, the solubility of THP itself may decrease upon cocrystallization.

#### Theophylline-Quercetin cocrystal

Quercetin (QUE) is a widely distributed bioflavonoid exhibiting a diverse range of pharmacological effects, including anti-inflammatory, antioxidant, anti-aggregatory, anticarcinogenic, anti-thrombotic, and vasodilatory properties. Notably, QUE demonstrates efficacy in controlling asthmatic reactions by suppressing the production of histamine and proteins while also inhibiting phospholipase A2 activity. However, QUE's classification as a BCS Class II drug with poor aqueous solubility (~ 2 μg/mL) [78], significantly restricts its clinical applications. A study by Lin Wang et al. [7], investigated cocrystallization as a strategy to improve QUE's solubility. Their findings revealed that both the hydrated (THP-QUE-H) and anhydrous (THP-QUE-A) cocrystals of THP and QUE exhibited significantly enhanced equilibrium solubilities compared to pure QUE. Specifically, the solubility of THP-QUE-H and THP-QUE-A reached 0.023 mg/mL and 0.031 mg/mL, respectively, representing approximately 4.6- and 6.2-fold increases compared to pure QUE·2H_2_O (0.005 mg/mL).

#### Theophylline-Baicalein cocrystal

Baicalein (BAI), a flavonoid compound extracted from the roots of *Scutellaria baicalensis*, possesses various therapeutic properties, including anti-inflammatory, anti-allergic, anti-viral, and anti-bacterial activities. However, its low aqueous solubility (16.82 μg/mL) significantly restricts its oral bioavailability and therapeutic efficacy. A promising strategy to overcome this limitation lies in cocrystallization. The study by Wen Li et al. [79] demonstrated that the THP-BAI cocrystal effectively enhanced the solubility and dissolution rate of BAI. In vitro dissolution tests were conducted in both hydrochloric acid (HCl, pH1.2) and phosphate buffer (PBS, pH6.8) to compare the dissolution profiles of baicalein, the THP-BAI cocrystal, and a physical mixture of THP and BAI. In HCl solution at pH1.2, the THP-BAI cocrystal exhibited a 2.2-fold increase in the dissolution rate compared to the physical mixture (53.6 ± 1.1% vs. 28.0 ± 4.6% after 360 min) and a significantly higher rate compared to the baicalein coarse powder (23.9 ± 1.6%). Similarly, in PBS solution at pH6.8, the THP-BAI cocrystal displayed a remarkable 7.1-fold enhancement in the dissolution rate (26.2 ± 5.9% after 360 min) compared to the baicalein coarse powder (3.7 ± 0.5%). The physical mixture showed a modest increase in dissolution rate (14.0 ± 1.3%) compared to the coarse powder. These findings highlight the potential of cocrystallization with THP as a valuable approach to improve the poor solubility of baicalein, thereby paving the way for enhanced oral bioavailability and therapeutic effectiveness.

#### Theophylline-Nicotinamide cocrystal

Nicotinamide (NCT), a member of the vitamin B family, exhibits polymorphism, with four reported polymorphs [80]. Notably, NCT can form a cocrystal with THP (THP-NCT cocrystal). Jie Lu et al. [37], investigated the THP-NCT cocrystal using solid-state grinding and slow evaporation from ethanol. Dissolution experiments were then conducted to compare the behavior of THP and the THP-NCT cocrystal. The findings revealed that the cocrystal exhibited superior solubility characteristics and dissolution rate compared to pure THP powder. At 25 °C, the THP-NCT cocrystal achieved a maximum concentration of 0.061 M within approximately 7 min, while THP powder reached its peak concentration of 0.056 M after about 8 min. Additionally, the concentration of THP-NCT in the dissolution medium consistently exceeded that of THP at all measured time points. For example, after 20 min, the concentrations of THP-NCT and THP were approximately 0.052 M and 0.034 M, respectively. These results suggest that cocrystallization with NCT offers a promising strategy to improve the dissolution profile of THP.

#### Theophylline-1,10-Phenathroline cocrystal and Na- (THP)_2_ClO·2H_2_O

Studies by Siphumelele Majodina et al. [64] investigated the cocrystallization of THP with 1,10-phenanthroline (Phen) and sodium ions, resulting in the formation of two hydrates: THP-Phen·2H_2_O and Na- (THP)_2_ClO_4_·2H_2_O. These cocrystals exhibited significantly improved solubility compared to their parent molecules. The solubilities of THP-Phen·2H_2_O and Na- (THP)_2_ClO_4_·2H_2_O were determined using high-performance liquid chromatography with ultraviolet detection (HPLC–UV) and compared to previously reported values for THP (8.3 mg/mL) and Phen (2.69 mg/mL). The results indicated a remarkable enhancement in solubility for both cocrystals: THP-Phen·2H_2_O reached 23.4 mg/mL, while Na- (THP)_2_ClO_4_·2H_2_O exhibited an even higher solubility of 32.2 mg/mL. Notably, Na- (THP)_2_ClO_4_·2H_2_O demonstrated the most significant improvement, with a solubility approximately four times greater than that of pure THP. In comparison, THP-Phen·2H_2_O displayed a tenfold increase in solubility compared to Phen and nearly threefold higher solubility than THP itself. X-ray diffraction analysis of the undissolved solids recovered from the solubility experiments confirmed that they matched the unit cells of the corresponding cocrystals (THP-Phen·2H_2_O and Na- (THP)_2_ClO_4_·2H_2_O), indicating their stability in water for a period of 24 h.

#### Theophylline-Favipiravir cocrystal

Favipiravir (Fav), commercially known as Avigan®, is a novel, potent antiviral drug that specifically inhibits the RNA-dependent RNA polymerase (RdRp) of RNA viruses [81]. A study by Poonam Deka et al. [46] explored the development of a drug-drug cocrystal of THP and Fav (Fav-THP) to improve the solubility of Fav. The anhydrous form of Fav exhibited a solubility of 2.94 mg/mL in distilled water and 7.83 mg/mL in pH7 phosphate buffer at room temperature (25°C). Notably, the Fav-THP cocrystal demonstrated enhanced equilibrium solubilities in both distilled water (3.84 mg/mL) and pH7 phosphate buffer (11.52 mg/mL) compared to the parent drug Fav. These findings suggest that cocrystallization with THP offers a promising strategy to improve the aqueous solubility of Favipiravir.

#### Theophylline-Flufenamic acid cocrystal

Flufenamic acid (FFA), an anthranilic acid derivative classified as a BCS Class II drug, exhibits analgesic, anti-inflammatory, and antipyretic properties [82]. Aitipamula et al. [17] investigated the cocrystallization of FFA with THP as a strategy to improve FFA's solubility. The cocrystal was prepared using two methods: slow solvent evaporation and solvent drop grinding. The study revealed that cocrystallization with THP significantly increased the solubility of FFA. Following crystallization, the solubility of FFA in pH 7.5 phosphate buffer with 1% tween 80 at 37 °C doubled compared to the uncomplexed drug after a one-day incubation period. Furthermore, the THP-FFA cocrystal exhibited an improved dissolution rate, suggesting a potential benefit for its oral bioavailability.

#### Theophylline-Niclosamide cocrystal

Sanphui et al. [22] employed the solvent drop grinding method to prepare cocrystals of THP with the active pharmaceutical ingredient (API) niclosamide. These cocrystals, named Niclosamide-Theophylline Acetonitrile Solvate (NCL-THP-CH_3_CN) or NCL-THP for short, and NCL-THP, were investigated for their potential to improve the solubility of niclosamide. Niclosamide is a highly effective antihelminthic drug used to treat worm infestations in both humans and animals. However, its classification as a BCS Class II drug necessitates formulation as a suspension due to its near-insoluble nature in water. Additionally, niclosamide exhibits limited solubility in various organic solvents such as diethyl ether, tetrahydrofuran, ethyl acetate, and dioxane. Powder dissolution experiments revealed that NCL-THP displayed the highest solubility and dissolution rate, reaching 223.1 mg/L after 2 h. While NCL-THP cocrystal exhibited a comparable dissolution rate to NCL-THP for the first 90 min, it achieved the second-highest solubility (181.2 mg/L) at the 2-h mark. In conclusion, both NCL-THP and NCL-THP cocrystal demonstrated significantly enhanced solubility compared to niclosamide alone. Notably, after 2 h, NCL-THP and NCL-THP were found to be 6.3 and 5.1 times more soluble than niclosamide, respectively.

#### Rucaparib-Theophylline monohydrate

Rucaparib (Ruc), an oral small-molecule poly (ADP-ribose) polymerase inhibitor (PARPi) approved by the U.S. Food and Drug Administration (FDA) and the European Medicines Agency (EMA), is used for the treatment of recurrent ovarian cancer [83] Currently marketed as immediate-release tablets containing rucaparib camsylate [84]. Ruc exhibits limitations in bioavailability and water solubility. To address these limitations and potentially reduce the required daily dosage without compromising efficacy, Mengyuan Xia et al. [71]. explored cocrystallization with THP as a strategy for improvement. Utilizing a liquid-assisted grinding method, they successfully produced rucaparib-theophylline monohydrate (Ruc-THP MH). Equilibrium solubility studies revealed that at pH2.0, 4.5, and 6.8, Ruc-THP MH exhibited significantly enhanced solubility compared to both rucaparib camsylate and the free drug base. Specifically, Ruc-THP MH demonstrated 3.0, 4.6, and 3.6 times higher solubility than rucaparib camsylate and 3.2, 4.6, and 3.6 times higher solubility than the free base, respectively. In powder dissolution experiments conducted at pH 4.5 and 6.8, Ruc-THP MH displayed the highest apparent solubility at 60 min, exceeding that of Ruc by approximately 2.2 and 3.0 times, respectively. Furthermore, the extended supersaturation of Ruc-THP MH observed at pH4.5 suggests a delayed precipitation and recrystallization process, potentially promoting improved drug absorption.

### Humidity stability

There is some inconsistency in the literature regarding the hygroscopicity of THP anhydrate and monohydrate with respect to relative humidity (RH). Certain studies have classified THP as a moisture-sensitive drug, capable of interconverting between crystalline anhydrate and monohydrate forms and readily absorbing water from the environment or during formulation processes [85]. Stephen A. Stanton and coworkers [86] asserted that THP exhibits high hygroscopicity despite its low aqueous solubility, leading to its designation as a last-resort treatment for patients unresponsive to conventional bronchodilators. However, Otsuka M et al. [87], produced two distinct types of THP anhydrate: Type I obtained through recrystallization from distilled water at 95°C and Type II obtained by dehydration of THP monohydrate. Their hygroscopicity studies revealed that at 35°C, Type I remained stable below 82% RH but transformed into the monohydrate form above 88% RH. Similarly, Type II exhibited stability below 66% RH but converted to the monohydrate form at 75% RH or higher. Andrew V. Trask et al. [18] further investigated the RH stability of THP anhydrate at four specific levels (0%, 43%, 75%, and 98% RH) across four time points (1 day, 3 days, 1 week, and 7 weeks). After 7 weeks, THP anhydrate maintained physical stability at 75% RH or below, while complete conversion to the monohydrate form occurred at 98% RH. These findings suggest that the hygroscopicity of THP anhydrate is relative. It demonstrates stability at low RH (around 66%) and can vary depending on the preparation method, with different sources exhibiting distinct hygroscopic properties. Due to its impact on storage conditions, solubility, and even biopharmaceutical properties, hygroscopicity is a critical consideration for THP. Consequently, researchers have explored the development of numerous THP cocrystals as a strategy to modulate the hygroscopicity of THP or its CCF at high humidity levels.

#### Theophylline-Apigenin cocrystal

Shan Huang and coworkers [88] obtained theophylline-apigenin (THP-AGN) cocrystal by slow evaporation. The water sorption and desorption processes of THP and THP-AGN were measured on an Intrinsic dynamic vapor sorption (DVS) instrument over a broad humidity range. The results showed that the THP-AGN cocrystal is much less hygroscopic than THP, even after exposure to high RH, and showed a greater resistance to hydration than THP alone.

#### Theophylline-Quercetin cocrystal

Humidity stability is a critical physicochemical property for pharmaceutical solid forms. The hydration or dehydration of these solids involves a phase transition between different hydrates (lower to higher or vice versa) or between an anhydrous and a hydrated form. This transition often comes with changes in the physicochemical characteristics of the solid forms. Anhydrous QUE, for example, is unstable to humidity, and the commercially available raw form is typically its dihydrate. In a study by Lin Wang et al. [7], the humidity stability of QUE was investigated under two different relative humidity conditions for 30 days. Their findings demonstrated that THP-QUE-A maintains its original solid phase even at 95% RH and 25 °C, while THP-QUE-H exhibits the same stability under both 95% and 15% RH. These results suggest that the two novel solid forms of THP and QUE exhibit superior phase stability against variations in relative humidity at ambient temperature. This makes them strong candidates for further development and study.

#### Theophylline-Favipiravir cocrystal

Poonam Deka and coworkers [46] reported a novel drug-drug cocrystal, THP-Fav, that exhibited improved moisture stability compared to the anhydrous form of THP, as evidenced by powder X-ray diffraction (PXRD) measurements. The estimated powder pattern of the THP-Fav cocrystal closely matched the PXRD pattern of a slurry sample, indicating that the cocrystal maintains its structure and does not dissociate into its individual components or convert to THP monohydrate. Furthermore, comparisons of PXRD patterns between the slurry residues and freshly prepared THP-Fav samples confirmed the high stability of the synthesized cocrystal. This stability is evident even under harsh conditions, such as exposure to a water slurry for approximately 18 h or a pH 7 phosphate buffer slurry for approximately 24 h.

#### Theophylline-Nicotinamide cocrystal

Jie Lu and coworkers [37] investigated the water sorption behavior of NCT, THP, and their cocrystals using dynamic vapor sorption and desorption isotherms. The THP-NCT cocrystal consistently adsorbed more water than THP and NCT at all RH levels. For instance, at 90% RH, the amount of water sorbed by THP-NCT, NCT, and THP were approximately 0.24%, 0.20%, and 0.023%, respectively. This observation suggests that the formation of the THP-NCT cocrystal significantly increases the hygroscopicity of theophylline.

#### Theophylline-Flufenamic acid cocrystal

Aitipamula and coworkers [17], employed a multi-pronged approach to evaluate the stability of the THP-FFA cocrystal and its parent molecules. This approach included slurry experiments at 37 °C, storage of the cocrystals at accelerated conditions, and using the DVS technique.

Slurry experiments using PXRD analysis demonstrated the stability of the THP-FFA cocrystal and FFA. No dissociation or chemical degradation of the cocrystal components was observed after slurrying in deionized water at 37 °C for 24 h. Conversely, THP transformed into its hydrate form under these conditions. Similarly, accelerated stability testing using PXRD revealed that both FFA and the THP-FFA cocrystal maintained their structural integrity after storage for 13 weeks at 40 °C and 75% RH. In contrast, THP readily converted to its hydrate form within just one day under these same conditions. DVS technology provided further insights into the moisture sorption behavior of these materials. Across a broad humidity range, both FFA and the THP-FFA cocrystal exhibited low moisture uptake (less than 2%). Notably, the absorption and desorption profiles for these materials closely overlapped, indicating a reversible sorption process. In contrast, THP displayed significant variations in its sorption patterns, along with a marked increase in moisture uptake as humidity levels rose. The significant moisture uptake (~ 9.5%) observed during the sorption profile of THP, coupled with the absence of weight loss during desorption, strongly suggests the formation of a THP hydrate.

### Biopharmaceutical activity enhancement

THP, a bronchodilator API, is also a frequently chosen CCF due to its Generally Recognized As Safe (GRAS) status. Consequently, THP cocrystals encompass both API-CCF and drug-drug cocrystal types. Notably, drug-drug cocrystals offer promising solutions to challenges often encountered in conventional combination therapy, such as solubility issues, variations in stability, and potential chemical interactions between the APIs. When two or more APIs cocrystallize, combination medications can be formulated for improved and more effective treatment of complex diseases where current monotherapies fall short of achieving the desired therapeutic outcome [20]. Numerous THP drug-drug cocrystals have been developed to enhance the physicochemical properties and further improve the biopharmaceutical activity of THP or its CCF. Examples of such THP cocrystals are provided below.

#### Theophylline-1,10-Phenathroline cocrystal and Na- (THP)_2_ClO·2H_2_O

Siphumelele Majodina and coworkers [64] investigated the in vitro antimicrobial susceptibility of a novel THP cocrystal with 1,10-phenanthroline (a pharmaceutically relevant CCF due to its antimicrobial activity) and a THP ionic cocrystal (sodium ion, Na- (THP)_2_ClO_4_·2H_2_O). The results indicated that both THP hydrate and Na- (THP)_2_ClO_4_·2H_2_O exhibited antibacterial and antifungal properties, particularly against drug-resistant human pathogens. These pathogens included Gram-negative bacteria (*A. baumannii, E. coli, K. pneumoniae*) and Gram-positive bacteria (*S. epidermidis*), as well as fungi (*C. albicans* and *C. tropicalis*). The good to moderate activity of THP within Na- (THP)_2_ClO_4_·2H_2_O against all investigated pathogens can be attributed to its high solubility and the combined effect of donor/acceptor sites from THP and the ClO^4−^ anion.

#### Rucaparib-Theophylline monohydrate

Rucaparib is a potent drug approved by both the U.S. FDA and the European Medicines Agency (EMA) for treating recurrent ovarian cancer. However, its clinical use is limited by its high variability in absorption (coefficient of variation: 54%) and a modest average absolute bioavailability of 36% [89, 90]. Mengyuan Xia et al. [71] investigated a Ruc-THP monohydrate cocrystal and demonstrated its potential to improve bioavailability in a pharmacokinetic study conducted with fasted female rats. The study revealed statistically significant differences in the maximum concentration (C_max_) and area under the curve (AUC_0–24h_) between the cocrystal and rucaparib camsylate at a single oral gavage dose of 63 mg/kg. Specifically, the Ruc-THP monohydrate exhibited 2.4-fold (90% CI: 2.1–2.9) higher C_max_ and 1.4-fold (90% CI: 1.2–1.5) greater AUC_0–24h_ compared to rucaparib camsylate.

#### Theophylline-Baicalein cocrystal

BAI, a widely used traditional Chinese medicine, possesses various pharmacological and therapeutic effects for treating hyperlipemia, hypertension, atherosclerosis, dysentery, common colds, inflammation, and even tumors [91, 92]. However, its low oral bioavailability significantly hinders its clinical efficacy. Wen Li and coworkers [79] successfully addressed this challenge by developing a THP-BAI cocrystal that demonstrably improves the oral bioavailability of BAI. A pharmacokinetic study in rats compared the plasma concentration and other pharmacokinetic parameters after oral administration of three formulations: BAI coarse suspension, a physical mixture suspension of BAI and THP, and a THP-BAI cocrystal suspension (all administered at the same BAI dose). The study revealed that the physical mixture suspension exhibited a 1.3-fold increase in AUC_0–t_ (area under the curve from time zero to infinity) compared to BAI alone (29.94 ± 5.43 h μg/mL vs. 23.02 ± 8.54 h μg/mL). However, the THP-BAI cocrystal demonstrated the most significant improvement, with an AUC_0–t_ of 147.59 ± 86.54 h· g/mL. This translated to a relative bioavailability that is 6.41 times greater (p < 0.05) than the BAI coarse suspension and 4.93 times higher (p < 0.05) than the physical mixed suspension.

#### Theophylline-Glibenclamide cocrystal

Glibenclamide (GCM), also known as glyburide, is a second-generation sulfonylurea medication administered orally for treating type II diabetes mellitus (non-insulin-dependent) [54]. According to the Biopharmaceutical Classification System, GCM falls under Class II due to its high permeability and low water solubility. Parnika Goyal et al. developed a novel THP-glibenclamide (THP-GCM) cocrystal using solvent-assisted grinding. In vivo studies demonstrated a significant improvement in the bioavailability of GCM through THP-GCM cocrystallization. The C_max_ of THP-GCM was approximately 1.6 times higher compared to pure GCM. Additionally, the time to reach maximum concentration (T_max_) was reduced from 240 min for pure GCM to 180 min for THP-GCM. These findings suggest that both the rate and extent of absorption were enhanced, indicating improved bioavailability. This improvement can translate to a faster therapeutic response, particularly at lower doses. Furthermore, the enhanced biopharmaceutical parameters of THP-GCM resulted in improved anti-diabetic activity, measured as the percentage of glucose reduction. The maximum percentage of glucose reduction after 4 h reached 53.68% for THP-GCM, compared to a maximum reduction of 40.68% for pure GCM.

#### Theophylline-Trimestic acid cocrystal

Cocrystallization with biologically relevant CCF offers a strategy to achieve more desirable physicochemical and biological properties for APIs. Olufunso O. Abosede et al. [93] demonstrated this concept by preparing a THP-trimesic acid (THP-TMA) cocrystal. This cocrystal exhibited improved water solubility compared to pure THP. Furthermore, the THP-TMA cocrystal displayed antimicrobial activity against various microorganisms, particularly clinically relevant Gram-negative bacterial human pathogens. In vitro antimicrobial assays revealed that the cocrystal can inhibit the growth of *A. baumannii, E. coli, K. pneumoniae*, and *P. aeruginosa*.

#### Theophylline-Apigenin cocrystal

The promising in vitro properties of the THP-Apigenin (THP-AGN) cocrystal, including improved solubility, intrinsic dissolution rate (IDR), and diffusion and flux profiles, prompted Shan Huang et al. [88] to further investigate its pharmacokinetic performance. Compared to the corresponding physical mixture and pure AGN, the cocrystal exhibited significantly better pharmacokinetic characteristics. Notably, the C_max_ (maximum plasma concentration) and AUC_0–24h_ (area under the curve from time zero to 24 h) of the THP-AGN cocrystal were markedly higher than those of AGN, with increases of 10.17 and 6.07 times, respectively. In contrast, the physical mixture of AGN and THP only displayed a 3.04-fold and a 1.76-fold increase in C_max_ and AUC_0–24h_, respectively, compared to pure AGN. The improved bioavailability of the THP-AGN cocrystal relative to pure AGN aligns well with its enhanced permeability, IDR, and solubility. Additionally, the significantly shorter T_max_ (time to reach maximum concentration) observed for the THP-AGN cocrystal indicates a faster arrival of peak plasma concentration, potentially leading to a more immediate therapeutic effect. Notably, the physical mixture of AGN and THP also exhibited increased C_max_ and AUC_0–24h_ compared to pure AGN, suggesting a possible synergistic effect of THP on the absorption of AGN.

## Conclusions

Theophylline readily forms cocrystals with a diverse range of CCF, including carboxylic acids, amides, lead compounds, and other drugs. These CCF typically possess good hydrogen bond acceptor and donor functionalities, such as the O=C and O–H groups of carboxylic acids, which can interact with theophylline to form cocrystals. Hydrogen bonding plays a dominant role in the formation of theophylline cocrystals. The imidazole ring of theophylline molecule acts as a potent hydrogen bond acceptor through its nitrogen atom (N) and oxygen atom (O). Additionally, the N–H group within the imidazole ring can function as a hydrogen bond donor, further facilitating interactions with CCF. Cocrystallization offers a powerful strategy to modify the physicochemical properties of theophylline or its CCF, ultimately leading to improved biopharmaceutical characteristics. Notably, theophylline can also interact with various metal ions, providing another avenue for tailoring its physicochemical properties. This review highlights several reported theophylline cocrystals, exemplifying the specific physicochemical properties that can be enhanced through cocrystallization and the subsequent improvement in their biopharmaceutical profiles.

The hygroscopicity of theophylline warrants further investigation and discussion. Existing literature reveals inconsistencies in the reported stability of theophylline at different relative humidity levels. Additionally, theophylline anhydrate prepared using various methods exhibits varying degrees of humidity stability. These observations necessitate further studies to definitively clarify the hygroscopicity of theophylline. When designing humidity stability experiments, careful consideration should be given to the preparation method of theophylline anhydrate and the specific polymorph (anhydrate or monohydrate) employed, as these factors can significantly influence the results.

Theophylline demonstrates the ability to cocrystallize with numerous cocrystal formers (CCFs) through a variety of common preparation methods. These methods include neat and liquid-assisted grinding, ball milling, isothermal slurry conversion crystallization, cooling crystallization, and solvent evaporation. The majority of theophylline cocrystals are obtained through mechanochemical processes, primarily neat and liquid-assisted grinding, as well as ball milling. Examples of such cocrystals include theophylline-quercetin, theophylline-oxalic acid, theophylline-glutaric acid, theophylline-malonic acid, and theophylline-maleic acid, among others. Single crystals of theophylline cocrystals are typically obtained through slow evaporation. When preparing theophylline cocrystals, it is crucial to consider key parameters specific to each method. For instance, in grinding processes, the strength and duration of grinding are important factors. In ball milling, the ball-to-material ratio is significant. For cooling crystallization, the cooling rate plays a vital role. In slurry conversion, the type, volume, and ratio of solvents can influence the formation of theophylline cocrystals. Following their formation, theophylline cocrystals are characterized using several common analytical methods. These include thermal gravimetric analysis (TGA), differential scanning calorimetry (DSC), Fourier transform infrared (FT-IR) spectroscopy, dynamic vapor sorption (DVS) analysis, and X-ray diffraction (XRD), among others.

While extensive research has been conducted on theophylline cocrystals, the focus has primarily been on their structure, the interaction between theophylline and CCF, and the improvement in physicochemical properties compared to the parent compounds. Studies investigating the biopharmaceutical properties of theophylline cocrystals are comparatively scarce. Cocrystallization of theophylline with certain lead compounds or marketed drugs has demonstrated the potential to enhance the bioavailability of these compounds or generate synergistic effects. This suggests that cocrystal formation can improve the drug-likeness of lead compounds and potentially alleviate adverse effects. To fully explore this potential, future research should prioritize pharmacokinetic, pharmacodynamic, and in vivo studies.

## Data Availability

The data supporting the findings of this study are available upon reasonable request from the corresponding author.
